# Modifiable hygiene practices and the burden of protozoal and helminthic infections among undernourished children in Taiz, Yemen: a hospital-based cross-sectional study

**DOI:** 10.3389/fpubh.2026.1896791

**Published:** 2026-08-12

**Authors:** Talal Alharazi, Jerold C. Alcantara, Rachel Hulse, Bandar Alharbi, Fawaz D. Alshammari

**Affiliations:** 1 Department of Medical Laboratory Science, College of Applied Medical Sciences, University of Hail, Hail, Saudi Arabia; 2 Department of Medical Microbiology and Immunology, Faculty of Medicine and Health Sciences, Taiz University, Taiz, Yemen; 3 Department of Medical Laboratory Science, Kasiska Division of Health Sciences, L.S. Skaggs College of Pharmacy and Health Professions, Idaho State University, ID, United States

**Keywords:** helminth infection, hygiene practices, intestinal parasitic infections, malnutrition, water sanitation and hygiene (WASH), Yemen

## Abstract

**Background:**

Intestinal parasitic infections and undernutrition create a combined health burden in children, especially in areas affected by long-lasting humanitarian crises. This study assessed the prevalence, types, magnitude of association and modifiable behavioral risks associated with intestinal parasitic infection in undernourished preschool children in Taiz, Yemen.

**Methods:**

A hospital-based analytical cross-sectional study was carried out from January 2025 to January 2026. The study involved 1,200 undernourished children aged 6 to 59 months who received care from general public hospitals and randomly selected private clinics and primary healthcare centers. A range of anthropometric measures were standardized to compute the World Health Organization *Z* scores. The detection of intestinal parasites was performed via direct wet–mount microscopic microscopy, the formal-ether concentration technique, modified Ziehl–Neelsen staining and the adhesive cellophane tape perianal swab for pinworm detection. Multivariable binary logistic regression was employed to assess the predictors of parasitic infection, taking into account sociodemographic characteristics.

**Results:**

The overall prevalence of IPIs was 38.5% (95% CI: 35.7–41.3%). *Entamoeba histolytica/dispar* and *Enterobius vermicularis* contributed the majority of the IPIs, with 14.5 and 11.5%, respectively. The nutritional status of infected children is significantly poorer than that of uninfected children, as shown by a lower mean weight-for-age Z score (−1.54 ± 0.82 vs. −1.11 ± 0.74, *p* < 0.001). Furthermore, a consistent pattern was also evident, with the IPI incidence increasing steadily from 33.3% in children with mild undernutrition to 79.2% in those with severe undernutrition (*p* < 0.001 for trend). Irregular nail trimming (aOR: 14.10; 95% CI: 10.15–19.55; *p* < 0.001) and drinking unfiltered water (aOR: 4.65; 95% CI: 3.10–6.95; p < 0.001) were identified as the most important modifiable independent risk factors in multivariable variable analysis. Infections also remained more common among females (aOR: 1.82, *p* < 0.001).

**Conclusion:**

Intestinal parasitic infections are prevalent in undernourished children and contribute to reduced anthropometric measurements. Strong, independent correlations with the drinking of untreated water and unhygienic nail cuttings revealed identifiable, modifiable behaviors that are important for health and development. The study data illustrate the need to integrate targeted hygiene and WASH programs and become standard in pediatric nutrition centers in unstable, low-resource contexts to curb fecal-oral disease transmission.

## 1 Introduction

Soil-transmitted helminth infections and enteric protozoal diseases (intestinal parasitic infections or IPIs) represent a substantial global health burden, particularly in low- and middle-income countries (LMICs) (1). Over 1.5 billion individuals are affected by soil-transmitted helminths, which, together with protozoan pathogens such as *Giardia lamblia* and *Entamoeba histolytica*, account for most cases of diarrheal illness in children (2, 3). The nature and level of disease transmission are influenced by socioeconomic conditions across Middle East and North Africa (MENA) countries. In Yemen, for example, the country has experienced civil unrest and armed conflict since 2015, causing catastrophic destruction of the national public health system, resulting in the breakdown of municipal water, sanitation and hygiene (WASH) services and creating a favorable atmosphere for the emergence and proliferation of enteric pathogens (4).

In Taiz Governorate, this public health crisis impacts individuals across all stages of life. Pregnant women in this area exhibit high infection rates of *Toxoplasma gondii* (5) and various intestinal parasites (6), which is largely attributable to contamination from unsafe water sources and intimate contact with animals. Similar patterns of widespread infestation are observed during childhood and adolescence among rural schoolchildren, who are frequently infected with intestinal protozoa and helminths, with a high prevalence of *Schistosoma mansoni* infection. This is caused by open defecation, contact with untreated water and poor personal hygiene (7, 8).

Intestinal helminthic or protozoal infections together with malnutrition in children are well described and viciously syndemic (9). Chronic undernutrition impairs mucosal and systemic immunity, increasing susceptibility to parasitic colonization (10), whereas enteric infections exacerbate malnutrition through enteropathy, malabsorption, nutrient sequestration, and chronic diarrhea (11). The vicious cycle between malnourishment and enteritis perpetuates growth stunting and has permanent effects on the physical growth of children as well as their cognitive development (11). Enteric pathogen control is therefore a necessary prerequisite for the successful rehabilitation of children suffering from severe malnutrition (12).

In settings prone to violence, a lack of resources and a severely damaged WASH system, the prevention of endemic infections primarily hinges on household hygiene behaviors (13). Because fecal-oral transmission is predominant for various protozoans, such as *Entamoeba histolytica*, and helminths, including *Enterobius vermicularis* and *Hymenolepis nana*, the ingestion of untreated water and insufficient handwashing dramatically increase disease risk (14). In particular, inadequate hand hygiene and infrequent fingernail trimming are increasingly recognized as major contributors to transmission because material under the nails can contain protozoal cysts and helminth eggs, promoting both self-infection and spread within households (15, 16).

Despite the well-established syndemic relationship between IPIs and pediatric malnutrition, facility-based epidemiological evidence from active conflict zones such as Taiz remains limited. While regional surveys have confirmed that IPIs are endemic (17), relatively few studies have measured this burden, especially among children with clinically defined undernutrition. In addition, much of the literature does not clearly separate the independent contributions of modifiable, household-level hygiene practices from broader socioeconomic factors. Recent reviews also note that many parasitology studies insufficiently control for socioeconomic confounders or for the continuous severity of anthropometric deficits, which weakens causal interpretation (18–22).

To address these critical knowledge gaps, the primary aim of this large, hospital-based cross-sectional study was to explicitly determine the prevalence and specific species distribution of IPIs among undernourished children under 5 years of age in Taiz, Yemen. The secondary aims were twofold: first, to assess the dose–response relationship between WHO-defined malnutrition severity strata and parasite positivity; and second, to identify independent, modifiable behavioral risk factors driving these infections.

## 2 Materials and methods

### 2.1 Study design and setting

A hospital-based analytical cross-sectional study was carried out from January 2025 to January 2026 to assess the prevalence of IPIs and examine their nutritional and behavioral associations among undernourished preschool children. This observational study was conducted in accordance with the Strengthening the Reporting of Observational Studies in Epidemiology (STROBE) guidelines (23).

The study was conducted in the governorate of Taiz in the highlands of southwestern Yemen, with altitudes varying between 1,200 and 2,800 meters. Taiz Governorate is the third most populous province of Yemen and has been severely affected by the worsening humanitarian situation that began in March 2015. The participants were drawn from several government general hospitals, private hospitals and health centers in Taiz city and adjacent areas. Since patients are referred from both urban and rural locations, these sites serve a broader socioeconomic and demographic mix of underfed children and the population in general, suggesting that the study population is a fair sample of underfed children in the governorate.

### 2.2 Study population and eligibility criteria

The study population included children aged 6–59 months who attended the participating health facilities and were identified as nutritionally compromised. Nutritional compromise was defined as a weight-for-age Z score (WAZ) of ≤ − 1 standard deviation from the WHO Child Growth Standards median or the presence of clinical signs of malnutrition as determined by the attending clinician. Children were eligible for inclusion only if their primary caregiver provided written informed consent and if a fresh stool sample appropriate for parasitological examination was provided.

**Figure 1:**
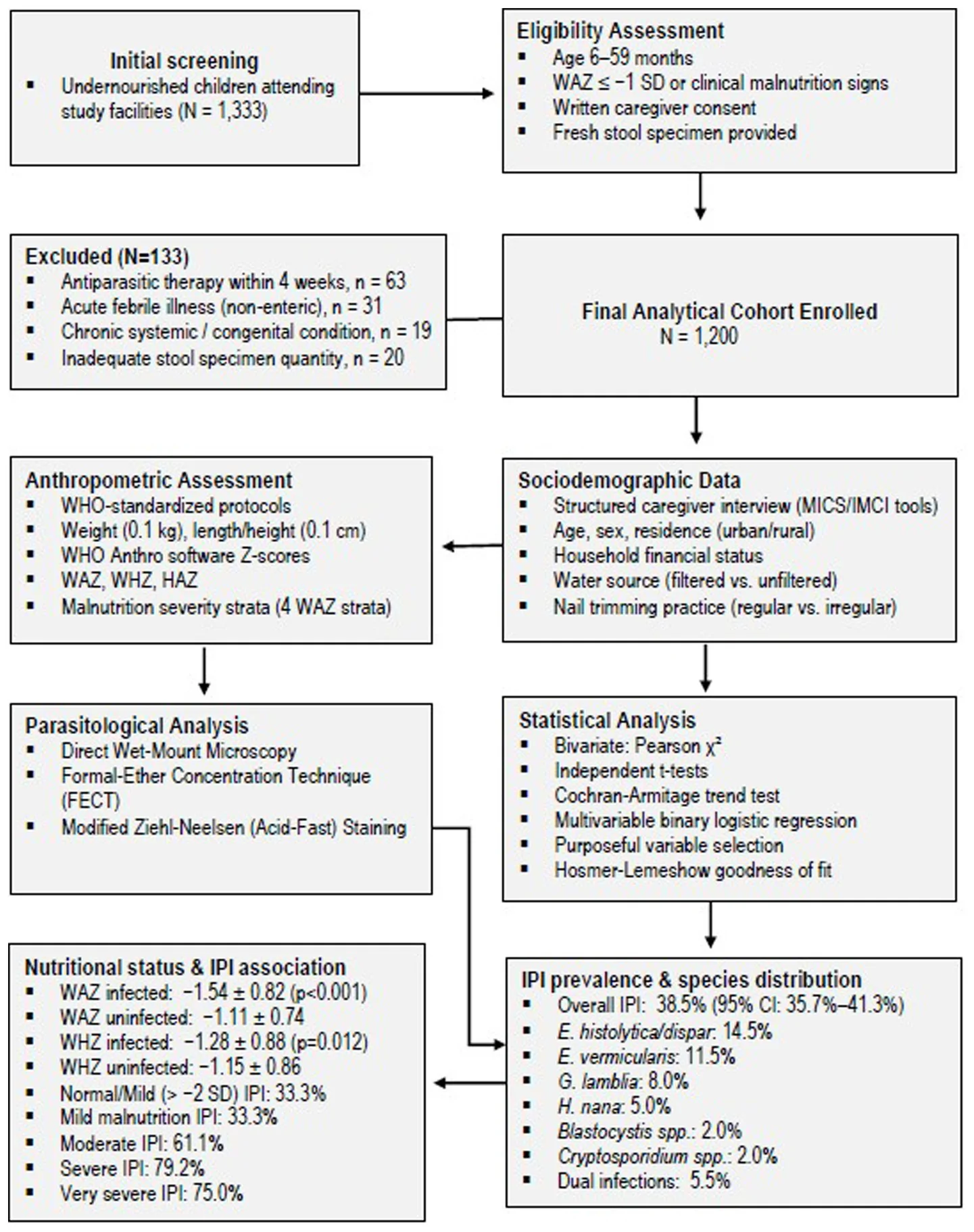
Study flow diagram.

To reduce confounding and misclassification, children were excluded if they (i) had taken antiparasitic drugs such as albendazole, mebendazole, or metronidazole within the previous 4 weeks; (ii) had an acute febrile illness due to a non-intestinal cause, such as malaria or acute lower respiratory tract infection; (iii) had a known chronic systemic illness or congenital condition that could independently affect nutritional status; or (iv) did not provide a stool sample of adequate quantity. A total of 1,333 children were screened; after 133 exclusions, 1,200 were included in the final analytical cohort (Figure 1).

### 2.3 Sample size determination and sampling strategy

The required sample size was calculated via the single population proportion formula: *n* = *Z*^2^(*α*/2) × *p*(1 − *p*)/*d*^2^. On the basis of a 95% confidence level (*Z* = 1.96), an expected IPI prevalence of 40% (*p* = 0.40), and a 5% margin of error (*d* = 0.05), the initial estimated sample size was 369 participants. Increasing this percentage by 10% for the nonresponse rate or unusable samples raised it to 406. To make the sample representative and increase the statistical power, 612 children who attended the triage units of the teaching hospitals were sampled via a consecutive sampling method and approached to enroll as they came into the triage units, whereas other children were enrolled via a convenient sampling method from three different centers, and all consecutive eligible children who attended the triage units were asked to be part of the study. A total of 1,200 undernourished children were included in the study, which is much greater than the desired sample size. A sample size of 1,200 resulted in >99% statistical power to detect the observed associations in the multivariable analyses, with a two-tailed *α*-level of 0.05.

### 2.4 Data collection procedures

With a structured, interviewer-administered questionnaire developed from the UNICEF Multiple Indicator Cluster Survey (MICS) and the WHO Integrated Management of Childhood Illness (IMCI) household tools, information was obtained on child sociodemographics (age, sex), household setting (rural vs. urban), economic status, water source (filtered vs. unfiltered), and nail hygiene of children (regular vs. irregular trimming). The questionnaire was validated by expert panels, including pediatric infectious disease specialists, a clinical nutritionist, and a field epidemiologist; the questionnaire was translated from English into Arabic and then back-translated into English. In a pilot study, 30 caregivers of children not included in the survey were tested, and interviews were held in private by trained field data collectors who used the Arabic dialect.

### 2.5 Anthropometric assessment and nutritional stratification

A standardized tool that adheres to WHO pediatric protocols was employed in the anthropometric evaluations. Body weight was assessed to the nearest 0.1 kg via infant or toddler scales, and the recumbent length (children aged 6–23 months) or standing height (children aged 24 months and above) was measured with standard infantometers or stadiometers to the nearest 0.1 cm, respectively. The raw anthropometric data were converted to WHO standard *Z* scores-weight-for-age (WAZ), height-for-age (HAZ) and weight-for-height (WHZ) via the WHO Anthro software package (24). Biologically implausible values were omitted. Nutritional deficits were classified as underweight (WAZ ≤ −2 SD), stunted (HAZ ≤ −2 SD) or wasting (WHZ ≤ −2 SD). Nutritional status by WAZ was divided into four levels: normal/mild (> − 2 SD), moderate (≤ − 2 to > − 3 SD), severe (≤ − 3 to > − 4 SD) and very severe (≤ − 4 SD).

### 2.6 Collection and parasitological examination of fecal samples

Caregivers received clean, tightly capped and leak-proof fecal collection containers, accompanied by detailed instructions on how to collect an approximately 5 g stool sample. The containers were transported to the central clinical laboratories within 2 h of collection to avoid any potential delays related to specimen fixation. After receipt at the laboratories, each sample was examined via three separate methods according to established World Health Organization (WHO) protocols for the parasitological examination of feces (25). Under direct wet mount microscopy, a small representative sample of each stool sample was mixed with a drop of 0.9% NaCl and with a drop of Lugol’s iodine solution separately on clean glass slides. The mixture was then covered with a coverslip and examined at ×100 and ×400 magnifications for the identification of protozoan trophozoites, cysts and oocysts and helminth eggs and larvae. To ensure the high reliability of the microscopic findings in the absence of digital imaging capabilities, a rigorous quality assurance protocol was implemented. All prepared slides were initially examined by trained laboratory technologists. Furthermore, 10% of all negative slides and 100% of positive or ambiguous slides were independently re-examined by a senior clinical parasitologist who was blinded to the initial findings. Any discrepancies were resolved by mutual consensus.

Another method employed was the formal-ether concentration technique (FECT). Approximately 1 g of stool was placed into a 15 mL centrifuge tube, mixed thoroughly with 10 mL of 10% formalin, poured through double-layer gauze into a 15 mL centrifuge tube, and centrifuged with diethyl ether at 500 × g for 1 min. The supernatant was discarded, and the sediment was examined microscopically under 400 × or 100 × wet-mount magnification. FECT improves the recovery rate of helminth eggs and protozoan cysts, with the absence of the required specimens in a wet mount or a low parasite load. Therefore, it was performed for each sample. Modified Ziehl-Neelsen (acid-fast) staining was also used on direct stool smears for Cryptosporidium oocysts. Because their morphology is not sufficient to be accurately identified by direct wet mounts or ether–formaldehyde concentrations, Cryptosporidium oocysts necessitate acid–fast staining for proper identification. Furthermore, to ensure the reliable detection of *Enterobius vermicularis*, the transparent adhesive tape (Scotch tape) perianal swab technique was utilized. This specialized technique is justified and strictly required because female pinworms primarily oviposit on the perianal folds rather than shedding eggs in the feces, causing standard stool concentration techniques to yield high false-negative rates. Caregivers were instructed to firmly press a strip of clear adhesive tape against the child’s unwashed perianal area immediately upon waking in the morning. The tape was subsequently smoothed onto a clean glass slide and examined microscopically at 100 × and 400 × magnifications.

### 2.7 Operational definitions of variables

Primary outcome: IPI status was considered positive if at least one species was identified and negative if no species was identified.

Water source: considered filtered if the water was processed through a filtration system (e.g., ceramic filter, slow sand filter), boiled or chlorinated and unfiltered if water was processed through a nonfiltration system (e.g., direct piped water access without filtration or untreated surface water).

Nail cuttings were considered to be cut regularly if the fingernails were cut at least once a week and cut irregularly if they were cut less frequently than once a week.

Household financial condition: classified as sufficient or insufficient, according to responses to whether families frequently spent enough to cover most basic food and nonfood expenditures.

### 2.8 Statistical analysis

Data were encoded twice into a password protected database, and a complete-case approach was used to handle missing values. All the statistical analyses were performed via IBM SPSS Statistics (version 26.0). Continuous variables were checked for normality and summarized as the mean ± standard deviation (SD). To evaluate unadjusted relationships between categorical predictors and IPI status, Pearson’s chi-square (*χ*^2^) test was employed. Student’s t tests for independent samples were used to compare the mean *Z* scores between infected and uninfected children. The Cochran–Armitage trend test was applied to examine the graded relationship between the prevalence of IPIs and the severity of malnutrition.

A multivariable binary logistic regression was performed to investigate independent determinants of IPIs. Variable selection was conducted via purposeful selection procedures (26). This study included age group, place of residence and financial household status as potential sociodemographic confounders. Behavioral hygiene variables with bivariate *p* values < 0.20 were introduced sequentially after sociodemographic factors were included in the multivariable model. The adjusted odds ratio (aOR) with a 95% confidence interval (CI) was used for measurement. A two-tailed p value < 0.05 was considered statistically significant.

### 2.9 Data privacy and clinical management

The information collected during the study was anonymized to protect the participants’ privacy. Any child identified as positive for an intestinal parasitic infection was referred to the respective pediatrician for appropriate medical management and received free antiparasitic medication according to national guidelines and WHO standards. In addition, all the participating children received tailored nutritional guidance and counseling.

## 3 Results

### 3.1 Baseline sociodemographic characteristics of the study cohort

The total number of undernourished children included in this cross-sectional study was 1,200. Most of the children were older than 2 years and up to 5 years (70.0%), and more males than females constituted the participants (57.0%). A highly vulnerable population represented the study group, as rural areas hosted most of the children (68.0%), and 85.0% belonged to a household with poor financial background. With respect to modifiable hygiene practices, 70.0% drank unfiltered water, and 45.0% consumed trimmed nails poorly. Table 1 shows the full baseline sociodemographic variables as well as the unadjusted bivariate associations of the IPIs.

**Table 1:** Baseline sociodemographic characteristics and crude bivariate associations with IPIs (*n* = 1,200).

Characteristic	Category	Total *N* (%)	Parasitized *n* (%)	Unadjusted OR (95% CI)/*p*-value
Age group	6 months–2 year	360 (30.0)	135 (37.5)	1.00 Reference
	> 2 year–5 year	840 (70.0)	327 (38.9)	1.06 (0.82–1.37)/*p* = 0.638
Sex	Male	684 (57.0)	220 (32.2)	1.00 Reference
	Female	516 (43.0)	242 (46.9)	1.86 (1.48–2.35)/*p* < 0.001*
Residence	Urban	384 (32.0)	142 (37.0)	1.00 Reference
	Rural	816 (68.0)	320 (39.2)	1.10 (0.85–1.42)/*p* = 0.443
Household financial status	Sufficient income	180 (15.0)	62 (34.4)	1.00 Reference
	Insufficient income	1,020 (85.0)	400 (39.2)	1.23 (0.88–1.72)/*p* = 0.227
Water source	Filtered water	360 (30.0)	50 (13.9)	1.00 Reference
	Unfiltered water	840 (70.0)	412 (49.0)	5.97 (4.31–8.29)/*p* < 0.001*
Nail trimming practice	Regular trimming	660 (55.0)	80 (12.1)	1.00 Reference
	Irregular trimming	540 (45.0)	382 (70.7)	17.53 (13.10–23.45)/*p* < 0.001*

*p < 0.05 statistically significant (two-tailed Pearson chi-square). OR, odds ratio; CI, confidence interval.

OR, odds ratio; CI, confidence interval. Data are expressed as n (column %). *p < 0.05 (two-tailed Pearson chi-square test).

### 3.2 Prevalence and species distribution of intestinal parasitic infections

The prevalence of intestinal protozoa and helminths (IPIs) among the study participants was 38.5% (95% CI: 35.7–41.3). Thirty-three percent (33%, *n* = 396) had a single-species infection, whereas only 5.5% (*n* = 66) of the cohort was affected by dual infections (only one type of polyparasitism was identified, Table 2). *E. histolytica/dispar* (14.5, 95% CI: 12.5–16.5%) was the most frequently identified organism in this study, followed by *E. vermicularis* (11.5, 95% CI: 9.7–13.3%**)** and *G. lamblia* (8.0, 95% CI: 6.5–9.5%). In comparison of parasite pairs, *E. vermicularis* together with *E. histolytica/dispar* was the most common dual infection encountered among the study participants (2.5%). This co-occurrence was most likely caused by the relatively high prevalence rates of these parasites and common transmission routes (fecal-oral) and less likely caused by a biological phenomenon increasing the susceptibility of host parasites to superinfection.

**Table 2:** Prevalence and species distribution of intestinal parasites (*N* = 1,200).

Infection/parasite	Prevalence (%)	95% CI (%)	*n*
Overall intestinal parasitic infections (IPIs)	38.5	35.7–41.3	462
Monoparasitism	33.0	30.3–35.7	396
Polyparasitism (dual infections only)	5.5	4.2–6.8	66
*Entamoeba histolytica/dispar*	14.5	12.5–16.5	174
*Enterobius vermicularis*	11.5	9.7–13.3	138
*Giardia lamblia*	8.0	6.5–9.5	96
*Hymenolepis nana*	5.0	3.8–6.2	60
*Blastocystis* spp.	3.0	2.0–4.0	36
*Cryptosporidium* spp.	2.0	1.2–2.8	24
Coinfection: *E. vermicularis + E. histolytica/dispar*	2.5	1.6–3.4	30
Coinfection: *E. histolytica/dispar + G. lamblia*	1.7	1.0–2.4	20
Coinfection: *E. vermicularis + G. lamblia*	1.3	0.7–1.9	16

### 3.3 Nutritional status and its association with intestinal parasitism

The extent of structural nutritional deficits throughout the cohort was characterized via baseline anthropometric assessments. Overall, 15.0% of the children were classified as underweight (WAZ ≤ −2 SD), 9.0% exhibited chronic malnutrition (stunting; HAZ ≤ −2 SD), and 8.0% suffered from acute malnutrition (wasting; WHZ ≤ −2 SD). Analysis of continuous WHO anthropometric *Z* scores demonstrated that parasitized children presented significantly more severe nutritional deficits than their nonparasitized counterparts did. The mean WAZ for parasitized children was significantly lower (−1.54 ± 0.82) than that for nonparasitized children (−1.11 ± 0.74; *p* < 0.001). Similarly, a statistically significant difference in the mean WHZ was detected between the infected and uninfected groups (−1.28 ± 0.88 vs. −1.15 ± 0.86, respectively; *p* = 0.012).

Crucially, a marked relationship was identified between malnutrition severity and IPI incidence. Infection positivity escalated progressively across WHO-defined WAZ severity strata: from 33.3% in children with normal/mildly impaired nutritional status to 61.1% in moderately malnourished children, peaking among those with severe (79.2%) and very severe malnutrition (75.0%) (*p* < 0.001 for linear trend) (Table 3).

**Table 3:** Anthropometric indices and malnutrition severity stratified by IPI status (*N* = 1,200).

Nutritional parameter	Total *N* (%)	Parasitized *n* (%)	Nonparasitized *n* (%)	*p*-value
Categorical indicators				
Underweight (WAZ ≤ −2 SD)	180 (15.0)	122 (67.8)	58 (32.2)	<0.001*
Stunting (HAZ ≤ −2 SD)	108 (9.0)	48 (44.4)	60 (55.6)	0.198
Wasting (WHZ ≤ −2 SD)	96 (8.0)	42 (43.8)	54 (56.2)	0.294
Malnutrition severity strata (WAZ)				
Normal/Mild (> − 2 SD)	1,020 (85.0)	340 (33.3)	680 (66.7)	<0.001*
Moderate (≤ − 2 to > − 3 SD)	108 (9.0)	66 (61.1)	42 (38.9)	
Severe (≤ − 3 to > − 4 SD)	48 (4.0)	38 (79.2)	10 (20.8)	
Very Severe (≤ − 4 SD)	24 (2.0)	18 (75.0)	6 (25.0)	
Continuous *Z* scores (mean ± SD)				
Weight-for-Age Z score (WAZ)		−1.54 ± 0.82	−1.11 ± 0.74	0.001*
Weight-for-Height Z score (WHZ)		−1.28 ± 0.88	−1.15 ± 0.86	0.012*
Height-for-Age Z score (HAZ)		−0.98 ± 0.91	−0.87 ± 0.88	0.081

Anthropometric categories are nonmutually exclusive. Categorical *p*-values by Pearson chi-square. Severity strata *p*-value (<0.001) represents the chi-square test for linear trend. Continuous *p*-values from independent-samples student’s *t*-tests. *p < 0.05.

WAZ, weight-for-age Z score; WHZ, weight-for-height Z score; HAZ, height-for-age Z score; SD, standard deviation. *p < 0.05.

### 3.4 Multivariable analysis of independent determinants of infection

Bivariate analyses indicated that age group, rural residency, and insufficient household income were not significantly associated with crude odds of acquiring an IPI (*p* > 0.05). Conversely, female sex, consumption of unfiltered water, and irregular nail trimming demonstrated strong unadjusted associations with parasitic infection.

To identify independent predictors of the IPI while controlling for potential structural confounders, a multivariable logistic regression model was constructed. Sociodemographic variables (age, residence, financial status) were forced into the model *a priori*, alongside behavioral variables, which had a bivariate *p* value < 0.20 (Table 4).

**Table 4:** Multivariable logistic regression analysis: Independent predictors of IPIs (*N* = 1,200).

Predictor variable	Category	aOR	95% CI for aOR	*p*-value
Age group	6 months–2 year (Ref.)	1.00	—	Reference
	>2 year–5 year	1.12	0.85–1.48	0.425
Sex	Male (Ref.)	1.00	—	Reference
	Female	**1.82**	1.38–2.40	<0.001*
Residence	Urban (Ref.)	1.00	—	Reference
	Rural	1.05	0.78–1.41	0.748
Household financial status	Sufficient income (Ref.)	1.00	—	Reference
	Insufficient income	1.18	0.82–1.70	0.371
Water source	Filtered water (Ref.)	1.00	—	Reference
	Unfiltered water	**4.65**	3.10–6.95	<0.001*
Nail trimming practice	Regular trimming (Ref.)	1.00	—	Reference
	Irregular trimming	**14.10**	10.15–19.55	<0.001*
Model fit statistics		Hosmer–Lemeshow: *χ*^2^(8) = 6.41, *p* = 0.602 c-statistic: 0.89 (95% CI: 0.87–0.91) VIF all < 1.40		

aOR, adjusted odds ratio; CI, confidence interval. Model fit: Hosmer–Lemeshow test, *χ*^2^(8) = 6.41, *p* = 0.602; c statistic = 0.89. *p < 0.05 (Wald chi-square test). Bold values indicate statistically significant findings.

After adjustment, three variables remained statistically significant independent predictors of the IPI. Compared with male sex, female sex was associated with an 82% increase in the odds of IPIs (aOR: 1.82; 95% CI: 1.38–2.40; *p* < 0.001). Unfiltered water consumption increased the odds of infection by almost five times (aOR: 4.65; 95% CI: 3.10–6.95; p < 0.001), highlighting the importance of having access to drinkable water. Finally, the most significant independent risk factor was irregular nail cutting, which increased the odds of IPI by 14 times (aOR: 14.10; 95% CI: 10.15–19.55; p < 0.001). This finding suggests a very actionable behavioral target for preventing fecal-oral transmission in this susceptible population.

## 4 Discussion

This hospital-based cross-sectional analysis of 1,200 undernourished children in Taiz Governorate offers significant epidemiological evidence. An overall IPI of 38.5% was found in this study, with waterborne protozoa being the most common pathogens. A critical finding of this study is the profound severity-dependent association between IPIs and continuous anthropometric indices, demonstrating that parasitized children presented significantly more severe nutritional deficits (lower mean WAZ and WH*Z* scores) than their uninfected peers. Furthermore, the high frequency of dual infections highlights a compounded burden of polyparasitism that likely accelerates this nutritional decline. According to the multivariable analysis, the strong modifiable determinants for IPIs were irregular nail trimming (aOR = 14.10) and drinking unfiltered water (aOR = 4.65). Taken together, these results underline how simple household behaviors contribute to maintaining the vicious cycle of infection leading to malnutrition in a region plagued by collapsed basic infrastructure.

### 4.1 Prevalence, epidemiological context, and species distribution

An IPI incidence of 38.5% indicates that Yemen is considered a high-burden country for intestinal parasitic infections, with a prevalence in a similar range to what recent pooled estimates reported among conflict-affected children who are involved in humanitarian crises (4). Prevalence values in this range are etiologically sound considering that the conflict began in 2015 and that the civil war has devastated municipal WASH infrastructure and sanitation systems and sustain fecal-oral transmission of IPIs (4).

The three major parasites encountered were *Entamoeba histolytica/dispar* (14.5%) and *Giardia lamblia* (8.0%), along with the self-transmitting autoinfective helminth *Enterobius vermicularis* (11.5%). These frequently found parasites are resilient to disinfection methods and are found to exist as cysts or eggs. Notably, water treatment systems undergo major ruptures in places where protozoan disease is endemic (27). However, without testing to identify parasitic antigens or conduct PCR testing, *E. histolytica* can be confused with nonpathogenic *E. dispar* on microscopic examination. Therefore, amoebiasis caused by *E. histolytica* might be overestimated in the study sample, indicating the need for molecular screening in the future.

### 4.2 Severity-dependent relationship between nutritional status and parasitism

Children infected with parasitic worms presented a lower mean WAZ and WHZ than did children not afflicted with parasitic worms. This result aligns strongly with scientific studies worldwide demonstrating that infections within the intestinal tract result in nutrient absorption difficulties, intestinal damage and inflammation throughout the body. Together, these symptoms interfere with proper physical growth (28).

Notably, there was a pronounced dose–response relationship between the severity of undernutrition and IPI incidence, increasing linearly from 33.3% (children with normal/mild undernutrition) to 79.2% (children with severe undernutrition), supporting the pathogenesis model of environmental enteric dysfunction (EED) (11, 12), whereby chronic exposure to parasitic and bacterial enteric pathogens injures the intestinal mucosa and suppresses the output of secretory IgA, increasing susceptibility to persistent colonization with such organisms and leading to malnutrition (by reducing nutrient digestion and absorption), growth faltering, increased risks of bacterial diarrhea and increased susceptibility to infections. The increasing load of intestinal pathogens also disturbs normal host metabolism of lipids and amino acids, possibly explaining the observed dose–response relationship, as demonstrated in recent studies (29, 30).

### 4.3 Behavioral and sociodemographic determinants of infection

#### 4.3.1 Female sex as an independent predictor

The 82% increased likelihood of developing IPIs in female children relative to male children (aOR: 1.82) requires careful interpretation. In contrast, other studies have shown that male sex is a risk factor for IPIs because, owing to the greater outdoor interaction of boys with contaminated soil, the higher IPI risk noted among girls in the Yemeni setting likely reflects local gender norms. Specifically, in a region where older sisters typically share domestic work involving the care of younger children and the management of waste, females might be at greater risk of acquiring infection due to the increased handling of wastewater and sewage (6, 31). Females often also stay indoors more and play with their children while remaining home; in so doing, they can pass infected persons to people with a greater number of contacts, including Enterobius autoinfection, while resting with their children in the shared bed (32).

Further research utilizing validated observational methods is essential to assess hand hygiene separately by sex so that any biologic predisposition can be distinguished from cultural biases and norms that contribute to increased risk. Direct observation remains the gold standard for assessment, although it has obvious limitations in terms of the reliability of measurements and the potential for observation reactivity bias (33). New electronic monitoring instruments can further decrease bias by offering objective, real-time results in addition to improving accuracy (33). Furthermore, research must work toward standardizing how water-related impacts are evaluated, as the absence of methods that consider gender suggests a crucial area that still needs to be explored (34).

#### 4.3.2 Unfiltered water consumption

The study revealed nearly 5-fold increased odds of IPIs for children drinking unfiltered water (aOR = 4.65, 95%). Protozoal cysts such as *Giardia* and *Entamoeba* are resistant to survival in aquatic settings and can be resistant to routine water treatment processes, thereby creating an ongoing contamination threat to drinking water (35). This association persisted among undernourished children and supports evidence from this region, where the leading cause of IPIs was identified to be drinking unsafe water from wells or streams in Taiz, Yemen (6). The substantial effect size persisted after adjusting for rural residence and household income, indicating that emergency point-of-use water purification remains an essential, standalone intervention to limit diarrheal infections in humanitarian contexts (13, 36).

#### 4.3.3 Irregular nail trimming as a principal risk factor

The most significant practical outcome observed in this study was the 14-fold elevated risk of IPI when nails remained untrimmed (aOR = 14.10). Fingernails have a propensity for trapping parasites beneath them, where parasitic ova can fester, especially for worms such as *E. vermicularis*, which spread easily through scratch-induced infections. These findings mirror conclusions drawn from other studies conducted in rural Taiz schools, where the independent predictors for high IPI levels, including schistosomiasis, were observed to be dirty, unwashed and untrimmed nails as well as unwashed hands (7, 8). Among people living in disadvantaged environments lacking basic WASH, nail dirtiness coupled with poor access to hygiene facilities and limited hand hygiene practices leads to major contamination and vulnerability.

### 4.4 Study strengths and limitations

The study has several strengths, including the use of a sufficiently powered and large sample size (*n* = 1,200), standardized calculation, and the use of WHO *Z* scores, with adjustment for numerous socioeconomic and nutritional variables in multivariate analysis, which yielded statistically robust associations that were also supported by cluster-robust sensitivity analyses. However, there are several limitations that should be acknowledged. The cross-sectional study design precludes the establishment of causality or the direction of the malnutrition-IPI association. The hygiene-related behavior data relied on self-reports by mothers (or caregivers), which is prone to social desirability bias, which is likely to attenuate the observed associations, so the true impact of poor hygiene might even be stronger than identified. Furthermore, the lack of use of Kato-Katz thick smear preparation likely undercounted the presence of STHs and mixed-worm infection. Hence, the reported prevalence of STH is likely to be a conservative estimate. Due to the severe infrastructural and equipment constraints inherent to this conflict setting, the clinical microscopes lacked digital imaging systems. Consequently, representative photomicrographs could not be captured for this study; however, rigorous blinded double-reading protocols by senior parasitologists were employed to maximize diagnostic credibility.

Additionally, parasite species identification relied solely on microscopic examination. In this severely resource-constrained environment, the routine implementation of confirmatory molecular techniques (e.g., PCR-based assays) or specific antigen detection methods was unfeasible. Consequently, morphologically identical species, such as pathogenic *E. histolytica* and non-pathogenic *E. dispar*, could not be differentiated, potentially leading to an overestimation of true amoebiasis. Therefore, any observed associations between amoebic infection and diarrheal symptoms must be interpreted with caution, as these symptoms could alternatively be driven by undetected viral or bacterial enteric pathogens co-endemic to the region. While appropriate for the clinical realities of a low-income setting, we strongly recommend the future incorporation of molecular diagnostics to improve species-specific diagnostic accuracy when resources permit.

## 5 Conclusions and public health implications

This study provides strong evidence of a dose–response syndemic relationship between intestinal parasitic infection and childhood undernutrition in conflict-affected regions of Yemen, which is especially concerning given that parasitic infections also commonly affect women in their reproductive years and school-aged children. The data imply that a potentially intergenerational fecal–oral transmission cycle is ongoing in Taiz, which faces a breakdown of communal sanitation, and that household hygiene practices independently influence the probability of infection. Regular clipping of fingernails or regular use of unfiltered tap water highlight key interventions that could address this situation at low cost. Given the findings of this syndemic, nutritional rehabilitation programs by incorporating hygiene educational modules for guardians, parents or caregivers and the provision of point-of-use water purification are urgently needed, as this disease will most likely persist into future generations.

## Data Availability

The original contributions presented in the study are included in the article/supplementary material, further inquiries can be directed to the corresponding author.
